# Gender relations, sexual behaviour, and risk of contracting sexually transmitted infections among women in union in Uganda

**DOI:** 10.1186/s12889-016-3103-0

**Published:** 2016-05-26

**Authors:** Olivia Nankinga, Cyprian Misinde, Betty Kwagala

**Affiliations:** Department of Population Studies, School of Statistics and Planning, College of Business and Management, Makerere University, P.O Box 7062, Kampala, Uganda

**Keywords:** Sexual empowerment, Partner behaviours, STIs, Uganda

## Abstract

**Background:**

Sexually transmitted infections (STIs) are a major reproductive and public health concern, especially in the era of HIV/AIDS. This study examined the relationship between sexual empowerment and STI status of women in union (married or cohabiting) in Uganda, controlling for sexual behaviour, partner factors, and women’s background characteristics.

**Methods:**

The study, based on data from the 2011 Uganda Demographic and Health Survey (UDHS), analysed 1307 weighted cases of women age 15–49 in union and selected for the domestic violence module. Chi-squared tests and multivariate logistic regressions were used to examine the predicators of STI status. The main explanatory variables included sexual empowerment, involvement in decision making on own health, experience of any sexual violence, condom use during last sex with most recent partner, number of lifetime partners and partner control behaviours. Sexual empowerment was measured with three indicators: a woman’s reported ability to refuse sex, ability to ask her partner to use a condom, and opinion regarding whether a woman is justified to refuse sex with her husband if he is unfaithful.

**Results:**

Results show that 28 % of women in union reported STIs in the last 12 months. Sexual violence and number of lifetime partners were the strongest predictors of reporting STIs. Women’s sexual empowerment was a significant predictor of their STI status, but, surprisingly, the odds of reporting STIs were greater among women who were sexually empowered. Reporting of STIs was negatively associated with a woman’s participation in decision-making with respect to her own health, and was positively associated with experience of sexual violence, partner’s controlling behaviour, and having more than one life partner.

**Conclusions:**

Our findings suggest that, with respect to STIs, sexual empowerment as measured in the study does not protect women who have sexually violent and controlling partners. Interventions promoting sexual health must effectively address negative masculine attitudes and roles that perpetuate unhealthy sexual behaviours and gender relations within marriage. It is also important to promote marital fidelity and better communication within union and to encourage women to take charge of their health jointly with their partners.

**Electronic supplementary material:**

The online version of this article (doi:10.1186/s12889-016-3103-0) contains supplementary material, which is available to authorized users.

## Background

Sexually transmitted infections (STIs) are a key reproductive and public health concern, especially in the era of HIV/AIDS. The World Health Organization (WHO) estimates that approximately 448 million infections occur worldwide, and about 47 % of them are among women [1]. In Uganda, the prevalence of STIs among women of reproductive age increased from 22 % in 2006 to 27 % in 2011 [2, 3]. The prevalence of STIs among women in union increased from 23 % in 2006 to 27 % in 2011. Women in union in this case means women who are either married or cohabiting. The Uganda AIDS Indicator Survey conducted in 2011 provided a higher estimate of women in union with STIs, at 37 %, a number that highlights the gravity of the situation in Uganda [4]. It is particularly important to note that in Uganda and elsewhere in sub-Saharan Africa, for instance in Zambia and Rwanda, the level of new HIV infections is higher among persons in union than in those not in union [2, 3].

Gender relations and sexual behaviours are pivotal in influencing sexual and reproductive health, as well as the general well-being of individuals and communities [5, 6]. Gender-based inequities have been associated not only with inequities in health but also with increased exposure to STIs [7]. Gender relations have a bearing on sexual behaviour, which in turn could determine one’s STI status. Socially constructed gender-based expectations define power relations, roles, obligations, and relationships between men and women [8]. Inequities in gender relations are often to the disadvantage of women, since women usually have a subordinate role in sexual relations [9].

Empowerment has been associated with improvement in health and development outcomes [10]. Empowerment is a process through which people gain control over their own lives. It is usually associated with an improved quality of life [11]. It is a multidimensional process through which persons lacking in certain resources or capabilities gain access to or control over those resources/capabilities. Empowerment relates to agency, whereby empowered persons are able to make strategic life choices (implying availability of alternatives) and can have the power to achieve their goals [12–16]. Sexual empowerment in this case primarily addresses issues associated with the individual woman and her interpersonal relationships with her partner. It mainly relates to “power within”—that is, self-confidence, a sense of self-worth and assertiveness, perception of the right to self-determination, and the confidence to act to attain the desired change in sexual relations. It also includes the “power to”—that is, having decision-making authority in sexual relations [17, 18]. In this paper, sexual empowerment mainly addresses the “power within” in relation to a woman’s perception of her ability to negotiate safer sex and the “power to” in relation to her participation in decision-making concerning her own health.

Women and/or their partners may engage in risky sexual behaviours that expose them to STIs. Contextual gender relations are important in influencing sexual behaviours, which include sexual and gender-based violence (SGBV), multiple sexual partnerships including polygyny, transactional sex, and unprotected sex [19–21]. Sexual behaviours are closely associated with a partner’s controlling behaviours, alcohol consumption, control over resources and household decision-making [5, 20, 22–25]. While fidelity is expected within marriage, marital partners may not be fully protected against STIs if either partner engages in risky sexual behaviours outside the union.

According to Part and colleagues [26], adherence to traditional gender roles related to sexual activity is stronger among females than males. Negotiating safer sex in such relationships is a challenge. Outside union, having trust in a relationship reduces the likelihood of condom use [27]. Within union, condom use is often resisted or not seen as necessary and is therefore limited [3, 28]. In most settings, faithfulness and trust are expected within marriage, and regular sexual activity is more or less deemed a right especially for the male partner [29].

STI and HIV infections among women are attributed to both biological and gender-related social factors. Women are biologically more prone to STIs, including HIV [30]. But women and adolescent girls also are disproportionately affected by STIs due to masculine ideals of risk taking, sexual conquest, and promiscuity [8, 31]. In Uganda, HIV prevalence is 8 % for women compared with 6 % for men [4].

It is assumed that addressing gender-based inequities in sexual relationships would mitigate effects on STIs [8]. However, although gender inequities have been associated with sexual ill health, the dynamics of relationships are more complex [32]. In Uganda, women’s empowerment with respect to household decision-making and attitudes towards violence was not a significant predictor of intimate partner sexual violence (IPV) [33]. Additionally, results of the Uganda AIDS indicator survey show that the prevalence of HIV was higher among employed women (9 %) than unemployed women (6 %) [4].

Studies have established that sexual and gender-based violence is associated with poor reproductive health outcomes, including STIs [32, 34–38]. The risk of contracting STIs is higher among women who experience sexual and gender-based violence (SGBV) [39]. Gender power imbalance, which is usually accompanied by partner abuse, increases the risk of STIs, including HIV [7, 8, 40]. Intimate partner violence (IPV) limits the possibility of negotiating for safer sex [41]. Perpetrators of IPV tend to engage in risky sexual behaviours that increase their partners’ risk of contracting STIs [42]. In Uganda, Koenig et al. [38] found that young women who reported that their first sex was coerced were less likely to use condoms or other modern contraceptives and more likely to report unwanted pregnancy and STI symptoms. Coercive sex is usually unprotected, thus exposing the victims to the risk of STIs [39, 43, 44]. It is important to note that directions of influence are not always consistent. IPV, for instance, could be both a cause and an effect of STIs, pointing to the cycle of violence [8]. A South African study found a positive association between a woman’s experience of domestic violence and her demand for condoms. The study also found that sexual control (empowerment) on the part of women was not directly associated with condom use [44, 45].

Controlling behaviours of male partners have been associated with violence [46]. As measured by demographic surveys, partners’ controlling behaviours, in the form of extreme possessiveness, jealousy, and attempts to isolate the spouse from their family and friends [3], were significant predictors of IPV in Uganda [25, 33] and of physical and sexual violence in Nigeria [20].

Excessive alcohol consumption, whether by men or women, induces risky sexual behaviour [47]. In Uganda and elsewhere, alcohol consumption has been associated not only with multiple sexual partnerships and non-use of condoms but also with STIs [8, 19, 26, 48, 49]. Other predictors of self-reported STIs among women are young age, high educational level, poverty, and concurrent, cross generational or multiple sexual partnerships [8, 50, 51]. A study in Uganda [52] found that marital status and having few sexual partners did not appear to protect young rural women from STIs, implying that male partners’ sexual behaviour may have an important impact on women’s risk of infection. Indeed, earlier studies of physical and sexual violence in Uganda highlighted the association between male partners’ risky (sexual) behaviours and STIs among women [19, 25].

Male circumcision has proved protective against HIV [53]. The prevalence of STIs has also been found to be lower among Muslims [53, 54], which may be due to the practice of circumcision among Muslims.

Individual empowerment does not occur in a vacuum. It is therefore important to consider contextual interpersonal and socio cultural factors [15]. Contextual, socio cultural, gender-based prescriptions and expectations with respect to sexual activity are likely to affect women in union. Analysis of the prevalence of STIs and the determinants of STI status among women in union is essential for a targeted response that will reduce STIs. No publication in Uganda has analysed the determinants of STI status, taking into account gender relations, in particular women’s empowerment, while adjusting for men’s controlling behaviours, sexual behaviours, and women’s background characteristics. This study, therefore, assessed the relationship between married women’s empowerment and their reported STI status while controlling for sexual behaviours, partner factors, and women’s background characteristics. With respect to gender relations associated with reproductive health, the International Conference on Population and Development (ICPD) in 1994, recognized that men have significant power or influence in most spheres of women’s lives. The ICPD also recognized the importance of improving communication between men and women in union on issues of sexuality and reproductive health and their joint responsibilities for better health outcomes [10]. This paper examines the association between women’s sexual empowerment and STI status.

## Methods

### Data source

This study used data from the 2011 Uganda Demographic and Health Survey (UDHS), which was conducted by the Uganda Bureau of Statistics and ICF Incorporation [55]. The dataset used is attached as Additional file 1. The UDHS is a national population-based household survey and uses a two-stage cluster sampling procedure. It included questions on demographic and socioeconomic characteristics of individuals, their sexual behaviour, gender relations and whether they had suffered from an STI in the 12 months prior to the survey [3].

The 2011 UDHS interviewed 8674 women age 15–49. Out of these, only women in union who were selected for the domestic violence module, a weighted sample of 1307 women were considered for this research.

In order to account for the complex survey design, data were weighted using the domestic violence variable (d005). The Stata survey command (svy) was used to apply the weights during analysis.

### Measures of the outcome variable

All the variables were extracted from the women’s individual record. The dependent variable “reported STI status” was captured using responses to any of the following three questions: whether respondents reported that in the last 12 months they (1) had a disease acquired through sexual contact; (2) had a bad-smelling abnormal genital discharge; or (3) had a genital sore or ulcer. Respondents who said yes to any or a combination of these three questions were recoded as having a reported STI, and a binary variable was constructed for this outcome (i.e., have STIs, or not).

### Measures of explanatory variables

The independent variables included responses to questions related to sexual behaviour; gender relations, which included sexual empowerment; partners’ behaviour; and background characteristics of women and their partners.

The variables concerning gender relations entailed analysis of women’s sexual empowerment and partners’ controlling behaviours. Indices were developed for each measure. Sexual empowerment included women’s responses to questions on the following: whether the woman can say no to a partner if she does not want to have sex; whether she can ask a partner to use a condom; and whether a woman is justified to refuse to have sex with her husband when she knows he has sex with other women. Women who responded yes to all the three questions were recoded as sexually empowered women.

Women were asked several questions concerning marital control by their partners. The index for partners’ controlling behaviour included women’s responses to questions addressing whether her partner (1) is jealous or gets angry if she talks to other men; (2) frequently accuses her of being unfaithful; and (3) insists on knowing where she is at all times. This variable was recoded into a dichotomous variable representing male partners who exhibit any of the three controlling behaviours versus partners who did not exhibit any of the controlling behaviours [20].

Household decision-making with respect to women’s own health was considered for analysis because it is closely associated with health outcomes. Women were asked “Who usually makes decisions about health care for yourself: you, your husband/partner, you and your husband/partner jointly, or someone else?” Women’s participation in decision-making included their individual or joint participation (with their partners). All other responses where women did not participate, namely partner alone, or other household members, were grouped in a single category of women who did not participate in decision-making concerning their health.

Experience of sexual violence is a binary aggregate variable that combines questions that asked women in union whether they have ever been: (1) physically forced into unwanted sex by a husband/partner; (2) forced into other unwanted sexual acts by a husband/partner; and (3) physically forced to perform sexual acts when you did not want to. “Don’t know” responses only totalled up to 7 (0.5 % of 1445 cases analysed). These cases could either be dropped or considered as a “yes” or “no”. Owing to stigma and secrecy associated with sexual abuse and sexual activity in general in the Uganda cultural context, cases of refraining from response are expected. Hence, “don’t know” responses were recoded as “yes.” These few cases have no significant impact on the results. The sexual behaviour measures included condom use during last sex with the most recent sex partner and the number of lifetime partners. Condom use was coded as either used a condom during last sex or no condom use. Three categories were recoded for number of lifetime partners: women with one lifetime partner, women with two lifetime partners, and women with three or more lifetime partners. Women who could not recall the number of partners were assumed to have more than two lifetime partners and were recoded in the third category—three or more lifetime partners.^1^

Women’s socio demographic factors considered were age, region, religion, household wealth quintile, and education. Spousal characteristics included age, education, occupation, and alcohol consumption. Age was grouped into four categories:15–19, 20–29, 30–39, and 40–49. Region was coded as Central, East, North, or West; religion as Catholics, Protestants, Muslims, or Pentecostals/Others. The category “Others” comprised smaller religious groups such as Seventh Day Adventists (SDAs). Wealth status was coded in quintiles: poorest, poorer, middle, richer, and richest. Education for both the respondent and the partner was coded as no education, primary education, or secondary or more. Women were asked how often their partners got drunk. Frequency of partner being drunk was grouped as never, often, or sometimes.

### Statistical analyses

As mentioned, only women in union who were selected and interviewed for the domestic violence module were included in the analysis. Data were analysed at the univariate, bivariate, and multivariate levels using the Stata software version 13.1. DHS used a two-stage stratified cluster sampling procedure. Sampling weights were calculated based on sampling probabilities for each sampling stage and for each cluster. The weights are applied to ensure representativeness of the study population [3]. In these analyses, we applied the domestic violence weights.

At the univariate level, descriptive statistics for the characteristics of the respondents and their spouses were presented, and, at the bivariate level, cross tabulations were used to determine the associations between the outcome variable [32] and background characteristics, sexual behaviour, spousal characteristics and behaviour, and sexual empowerment variables. Multiple logistic regression models were fitted to determine the relationship between predictors and the reported STI status. Adjusted odds ratios and 95 % confidence intervals of predictors were reported. The models were fitted in three steps: Model I contained the main predictor variable namely sexual empowerment to assess whether it independently predicted women’s STI status. Model II contained the main predictor variable and added other important explanatory variables i.e. sexual behaviours, woman’s involvement in decision-making concerning her own health, and partner control behaviours. Model III contained all explanatory variables controlling for women’s background characteristics.

### Ethical considerations

The paper is based on data in the public domain. Permission to use the data was obtained from the DHS program. The survey adhered to the World Health Organization’s ethical and safety recommendations for research on domestic violence. Informed consent was obtained from participants and their participation was on voluntary basis. For purposes of maintenance of anonymity, participants’ identifiers were not included in the dataset [3]

## Results

### Distribution of respondents by background characteristics

Table 1 presents descriptive results of the analysis. Out of the study sample of 1307 women in union who were selected for the domestic violence module, 27 % reported an STI or STI symptoms in the last 12 months. Over half (54 %) were sexually empowered, and 59 % were involved in decision-making concerning their own health. About one-quarter (27 %) of the women reported experience of intimate partner sexual violence. Only 9 % of the women used a condom with the most recent partner. Over half (53 %) of the women reported more than one lifetime sexual partner. With respect to socio demographic factors, the highest proportion of women were Catholic (40 %); age 20–29 (46 %), and with primary or no formal education (77 %). About three-quarters of the women’s partners (72 %) exhibited controlling behaviours, while 60 % never consumed alcohol.

**Table 1:** Percentage distribution of respondents by gender relations, sexual behaviour, partner factors, and women’s background characteristics

Variables	Freq. (1307)	Percent
Had STI in last 12 months		
No	949	72.6
Yes	358	27.4
Sexual empowerment		
Not empowered	601	46.0
Sexually empowered	706	54.0
Involvement in decision making on own health		
Not involved	532	40.8
Involved	774	59.2
Experience of any sexual violence		
No	950	72.8
Yes	356	27.3
Condom used during last sex with most recent partner		
No	1189	91.0
Yes	118	9.0
Number of lifetime partners		
One	612	46.8
Two	378	29
Three or more	316	24.2
Partner control behaviours		
Not controlled	364	27.9
Controlled in one or more	942	72.1
Women’s age group		
15–19	108	8.2
20–29	597	45.7
30–39	392	30.0
40–49	210	16.1
Women’s education		
None	222	17.0
Primary	785	60.1
Secondary +	299	22.9
Wealth index		
Poorest	243	18.6
Poorer	260	19.9
Middle	262	20.1
Richer	255	19.5
Richest	286	21.9
Religion		
Catholic	527	40.4
Protestant	373	28.5
Muslim	176	13.5
Pentecostal/others	231	17.7
Region		
Central	366	28.0
East	344	26.3
North	251	19.2
West	346	26.4
Partner’s education level		
None	132	10.1
Primary	706	54.0
Secondary +	469	35.9
Partner’s alcohol consumption		
Never	782	59.9
Often	199	15.2
Sometimes	326	24.9

### Association between STI status and independent factors

Bivariate results show that the association between women’s sexual empowerment and STI status is significant. Other indicators of women’s status showing significant associations with STI status at the bivariate level include women’s participation in decisions about their own health, experience of sexual violence, number of lifetime partners, and partner control behaviours (Table 2). Table 2 further shows that among other factors, wealth status, religion, and region were significantly associated with STI status.

**Table 2:** Percentage of women who reported STIs or STI symptoms by selected gender relations, sexual behaviour, partner factors, and women’s background characteristics

Variables	Percent of women reporting STIs	95 % CI	*P* value
Sexual empowerment			0.008
Not empowered	22.8	18.7–27.4	
Sexually empowered	31.3	26.8–36.2	
Involvement in decision making on own health			0.002
Not involved	33.1	27.7–38.8	
Involved	23.5	20.2–27.2	
Experience of any sexual violence			
No	22.6	19.0–26.7	
Yes	40.1	34.1–46.3	
Number of lifetime partners			0.000
One	18.4	14.9–22.4	
Two	28.7	22.3–36.2	
Three or more	43.2	36.4–50.2	
Partner control behaviours			0.000
Not controlled	17.7	13.3–23.1	
Controlled in one or more	31.1	27.3–35.2	
Condom used during last sex			0.123
No	26.2	23.2–29.4	
Yes	39.5	23.4–58.2	
Women’s age			0.572
19–19	23.0	15.3–33.1	
20–29	26.2	21.4–31.7	
30–39	30.2	24.8–36.2	
40–49	27.7	20.9–35.6	
Women’s education			0.454
None	21.2	16.2–29.5	
Primary	28.7	25.0–32.7	
Secondary +	27.8	18.8–39.2	
Wealth index			0.007
Poorest	19.0	14.0–25.2	
Poorer	22.2	17.4–27.8	
Middle	35.3	26.3–45.5	
Richer	33.1	26.0–41.1	
Richest	26.8	21.1–33.5	
Religion			0.014
Catholic	21.9	17.0–27.7	
Protestant	27.3	22.3–32.9	
Muslim	36.5	28.5–45.5	
Pentecostal/others	33.0	24.7–42.4	
Region			0.000
Central	31.9	26.7-37.5	
East	31.3	25.7-37.6	
North	10.5	7.4-14.7	
West	31.0	23.1-40.2	
Partner’s alcohol consumption			0.556
Never	28.9	25.1–33.0	
Often	24.2	18.3–31.4	
Sometimes	25.6	17.6–35.8	
Partners education level			0.206
None	18.8	11.8–28.67	
Primary	28.4	24.5–32.6	
Secondary +	28.3	22.6–34.7	

All the background factors analysed at the bivariate level were included in the final model with the exception of the respondent’s education and her partner’s education. Wealth status and partner’s education highly correlated with women’s education. Of the three variables, we opted to retain wealth status, which was significantly associated with sexual empowerment at the bivariate level of analysis.

### Adjusted associations between Women’s sexual empowerment, sexual behaviour, and partner behavioural factors and STI status

Multiple logistic regression models were fitted to establish the association between women’s sexual empowerment and reporting of STIs, controlling for sexual behaviour, partner characteristics and behavior, and women’s background characteristics. The logistic models were fitted in three steps: Model I contained only sexual empowerment; Model II added sexual behaviours, woman’s involvement in decision-making concerning her own health, and partner control behaviours, and Model III added women’s background characteristics, as presented in Table 3.

**Table 3:** Adjusted odds ratios (AORs) for reporting STIs among women in union in Uganda

	Model I		Model II		Model III	
	Odds ratio	[95 % CI]	Odds ratio	[95 % CI]	Odds ratio	[95 % CI]
Sexually empowered (ref: no)						
Yes	1.54**	1.12–2.13	1.38*	1.01–1.88	1.42*	1.01–1.92
Involvement in decision making on own health (ref: not involved)						
Involved			0.68*	0.50–0.93	0.70*	0.51–0.96
Condom use (ref: no)						
Yes			1.66	0.77–3.56	1.71	0.86–3.38
Number of lifetime partners (ref: one)						
Two			1.52*	1.01–2.29	1.51*	1.02–2.21
Three or more			2.77***	1.92–4.01	2.62***	1.73–3.99
Experience of any sexual violence (ref: no)						
Yes			2.12***	1.52–2.96	2.11***	1.48–3.02
Partner control behaviour (ref: no control)						
Yes			1.56*	1.08–2.26	1.69**	1.16–2.48
Partner’s frequency of being drunk (ref: never)						
Often			0.66	0.42–1.02	0.78	0.48–1.27
Sometimes			0.86	0.54–1.36	0.91	0.58–1.44
Wealth Index of respondent (ref: poorest)						
Poorer					1.09	0.64–1.83
Middle					1.64	0.93–2.89
Richer					1.35	0.81–2.25
Richest					0.84	0.48–1.49
Age of respondent (ref: 15–19)						
20–29					0.84	0.58–2.08
30–39					1.10	0.72–2.65
40–49					1.38	0.62–2.39
Religion (ref: Catholic)						
Protestant					1.41	0.92–2.14
Muslim					1.80*	1.12–2.88
Pentecostal & others					1.47	0.91–2.36
Region (ref: Central)						
East				0.82	0.82	0.54–1.27
North				0.37	0.37***	0.22–0.63
West				1.25	1.25	0.75–2.10

*CI* Confidence Interval, *Ref* Reference Category, *p < 0.05- **p < 0.01- ***p < 0.001

Sexual empowerment independently predicted STI status and remained significant after controlling for other independent variables in the subsequent two models, although the p-value in model III was marginal (*p*-value 0.044). On average, compared with women who are not sexually empowered, the odds of reporting STIs were higher among sexually empowered women (AOR = 1.42; CI 1.01–1.92). Women’s involvement in decision-making concerning their own health was also significantly associated with STI status. On average, the odds of reporting STIs were lower among women who participated in decision-making (individually or jointly with their partners) concerning their own health (AOR = 0.69; 95 % CI 0.50–0.96). Most of the sexual and partner behavioural factors, namely number of lifetime partners, experience of sexual violence and partners’ controlling behaviours, significantly predicted STI status. On average, the odds of reporting STIs were higher among women with experience of sexual violence (AOR = 2.11; 95 % CI 1.48–3.02), women with controlling partners (AOR = 1.69; 95 % CI 1.16–2.48), and women with two, three, or more lifetime partners (AOR = 1.51; 95 % CI 1.02–2.21and AOR = 2.62; 95 % CI 1.73–3.99, respectively).

The odds of reporting STIs were also higher among Muslim women compared with Catholic women (AOR = 1.80; 95 % CI 1.12–2.88), and lower among women in the Northern region compared with those in the Central region (AOR = 0.37; 95 % CI 0.22–0.63). Condom use, partner’s alcohol consumption, women’s level of education, and women’s age were not significantly associated with STI status.

## Discussion

Significant predictors of reporting STIs among women in union in Uganda were sexual empowerment, participation in decision-making on own health, experience of intimate partner sexual violence, partner’s control behaviours, number of lifetime partners, religion, and region. Results on the association between sexual empowerment and STI status suggest an opposite relationship to what we expected. Women’s sexual empowerment had marginal significance and does not appear to protect women in union from the risk of contracting STIs, controlling for sexual behaviour, experience of sexual violence, partner control behaviour, and background factors considered in the model.

It is evident that sexual empowerment, as measured by a woman’s reports regarding her ability to say no to her partner if she does not want to have sex, whether she can ask her partner to use a condom, and whether she is justified to refuse sex with her husband when she knows he has sex with other women, does not necessarily translate into protection against STIs. Effecting one’s desires in a marital relationship requires cooperation between partners. Additionally, couples rarely disclose their extramarital sexual activities to each other [56–58], which can expose them to greater risk of contracting STIs. As our findings show, this is particularly a challenge in the context of sexual violence perpetrated by women’s partners. It is important to note that survey questions related to sexual empowerment addressed opinions and possibilities that may not always translate into practice, owing to spousal and contextual factors [15]. Our findings support findings of studies elsewhere in Sub Saharan Africa which show that within marriage, indefinite/consistent condom use and abstinence, are practically impossible due to expectation of childbearing, relationships based on trust, and possible lack of knowledge of either partner’s extramarital sexual activities [56–59]. Ability to negotiate safer sex with a partner in union is challenged by fear of encouraging extra marital sexual relationships, plus sexual, physical and economic violence [59]. There is evidence that a woman’s interest in condom use in a committed relationship is associated with perceived need for protection against STIs [58]. This implies that such women are more vulnerable to STIs and could explain the higher likelihood of reporting STIs by such women. However, it was not possible to establish whether a woman’s ability to ask for a condom was a result of a high STI risk perception.

In contrast, women’s participation in decision-making concerning their own health, either individually or jointly with their partners had a mitigating effect on STIs. Studies specifically addressing the association between women’s autonomy with respect to decision making concerning their health and STIs are rare. However, studies addressing different reproductive health outcomes reveal a similar pattern. These studies show a positive association between decision making autonomy in general and contraceptive use [60], ante natal and skilled delivery care [61]. The survey question addressing participation in decision-making asked about actual situations in which women could make choices [12].

In our study of Uganda, as established elsewhere [32, 34–38], intimate partner violence (IPV) was the strongest predictor of STI status. IPV indicates poor conjugal relationships and lack of self-control, often accompanied by extramarital relations. It is also an indicator of lack of empowerment for women who are victims of this violence. As noted earlier, it is a challenge to negotiate for safer sex in the context of such violence. As Carpenter and colleagues have noted [31], male partners were twice as likely to be the source of HIV infection compared with their female counterparts. The situation is compounded by gender roles that promote female subordination in sexual relationships [9].

Closely related to sexual violence against women are controlling behaviours by their partners. For this paper, these included partner’s jealousies, accusations of unfaithfulness and knowing where the woman is at all times. These are important indicators of partners’ insecurities, abusive and risky behaviours, and possible lack of empowerment for women [20, 25, 33, 46]. Such behaviours increase the risk of STIs [42].

Among men, sexual activity with many partners increases the odds of STIs [31]. This is also the case among women with more than one lifetime partner [62]. Closely related is religion, where our study found that Muslim women had increased odds of reporting STIs. Although Muslim women could be better protected, assuming that they are in union with Muslim men, where male circumcision should have mitigating effects on STIs [53, 54], it is evident that they are not fully protected. The increased odds of Muslim women reporting STIs compared with other religions in our study may be explained by the high practice of polygamy among Ugandan Muslims. Polygamy among Muslim women in Uganda stands at 42 % compared with 28 % nationally [2, 3]. This area requires further research.

This study was unable to exhaustively analyse the effect of women’s risky sexual behaviours on their STI status because variables such as transactional sex were not captured. A better index of sexual empowerment would have been possible from survey questions addressing respondents’ actual practices [12, 17, 18] rather than possibilities or opinions. DHS data are limited in their ability to measure processes such as empowerment. Also, although they are nationally representative, DHS data are cross-sectional and thus cannot determine causal relationships. It was not possible to determine the source of STIs reported by women, although many studies associate STIs with male partner’s risky sexual behaviour. Nevertheless, our study provides important insight into determinants of STIs among women in union that could be the basis for programmatic response. Our findings make a vital contribution to the understanding of the risks and benefits of empowerment within union.

## Conclusions

Sexual empowerment was significantly associated with STI status, but odds of reporting STIs were higher among sexually empowered women, although with marginal statistical significance. Women’s participation in decision-making concerning their own health reduced the odds of contracting STIs. The strongest predictors of reporting STIs, namely sexual violence and number of lifetime partners, are direct risk factors of STIs. Reporting of STIs was also positively associated with partners’ controlling behaviours. With respect to STIs, sexual empowerment does not appear to protect women in union who have violent, controlling partners. Sexual empowerment is also not protective where a woman had more than one lifetime partner.

Interventions promoting sexual health must effectively address negative masculine and feminine attitudes and roles that perpetuate unhealthy sexual behaviours and relations within union. Persons in union are equally at risk of STIs if either partner engages in risky sexual behaviours. It is therefore important to promote fidelity and better communication between partners in union [10], and where necessary to encourage regular testing and treatment of STIs. Women need to take charge of their own health jointly with their partners.

## Additional file

Additional file 1: Dataset. (SAV 40698 kb)

## Abbreviations

AIDS: acquired immune deficiency syndrome
DHS: demographic and health survey
HIV: human immunodeficiency virus
ICPD: International Conference on Population and Development
IPSV: intimate partner sexual violence
IPV: intimate partner violence
SDA: Seventh Day Adventist
SGBV: sexual and gender based violence
STI: sexually transmitted infections
SVY: the survey prefix command that takes the sample design into account when calculating standard errors
UBOS: Uganda Bureau of Statistics
UDHS: Uganda Health and Demographic Survey
USAID: United States Agency for International Development
WHO: World Health Organization

## Footnotes

1 The two missing cases for this question were dropped.
